# Supplementary material for: The Tripartite Lichen Ricasolia virens: Involvement of Cyanobacteria and Bacteria in Its Morphogenesis
Source: Microorganisms. 2023 Jun 7;11(6):1517. doi: 10.3390/microorganisms11061517 (PMC10305269; doi:10.3390/microorganisms11061517)
Supplement: Supplementary file 1 [file microorganisms-11-01517-s001.zip › microorganisms-2403179-Supplementary Figures.pdf]

Ricasolia virens documents. Supplementary Figures

Figure S1

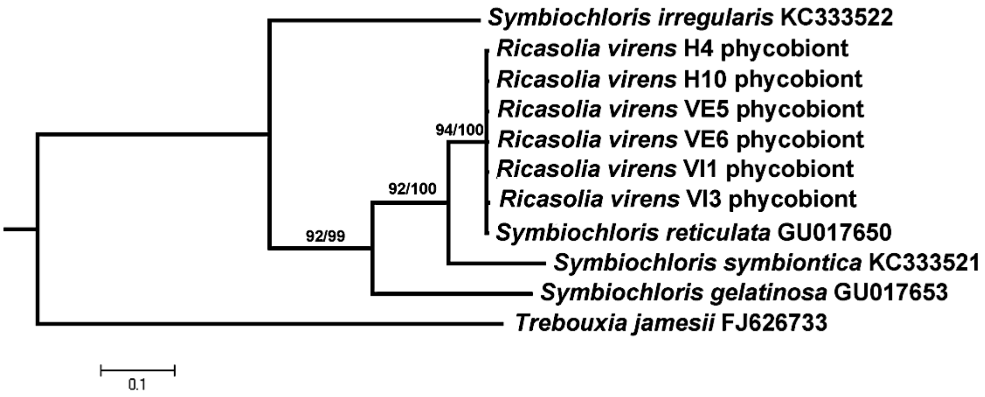

Figure S1. Phycobiont phylogenetic analysis. Rooted gene tree representing sequences of *Symbiochloris* retrieved from the GenBank and six newly generated sequences (VI1-3, VE5-6, H4-10). Values at nodes indicate statistical support estimated by two methods: posterior probabilities (PP, MrBayes analysis) and bootstrap support (BS, RAxML analysis).

Figure S2

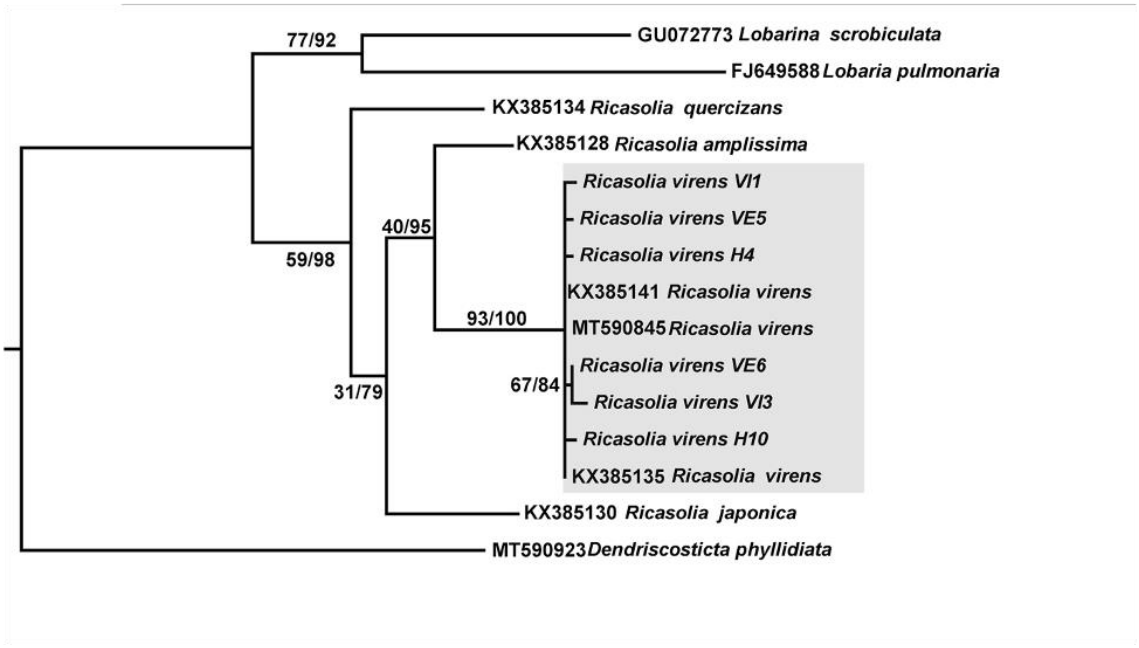

Figure S2. Mycobiont phylogenetic analysis. Rooted gene tree representing sequences of *Lobarina*, *Lobaria* and *Ricasolia* retrieved from the GenBank and six newly generated sequences (VI1-3, VE5-6, H4-10). Values at nodes indicate statistical support estimated by two methods: posterior probabilities (PP, MrBayes analysis) and bootstrap support (BS, RAxML analysis).

Figure S3

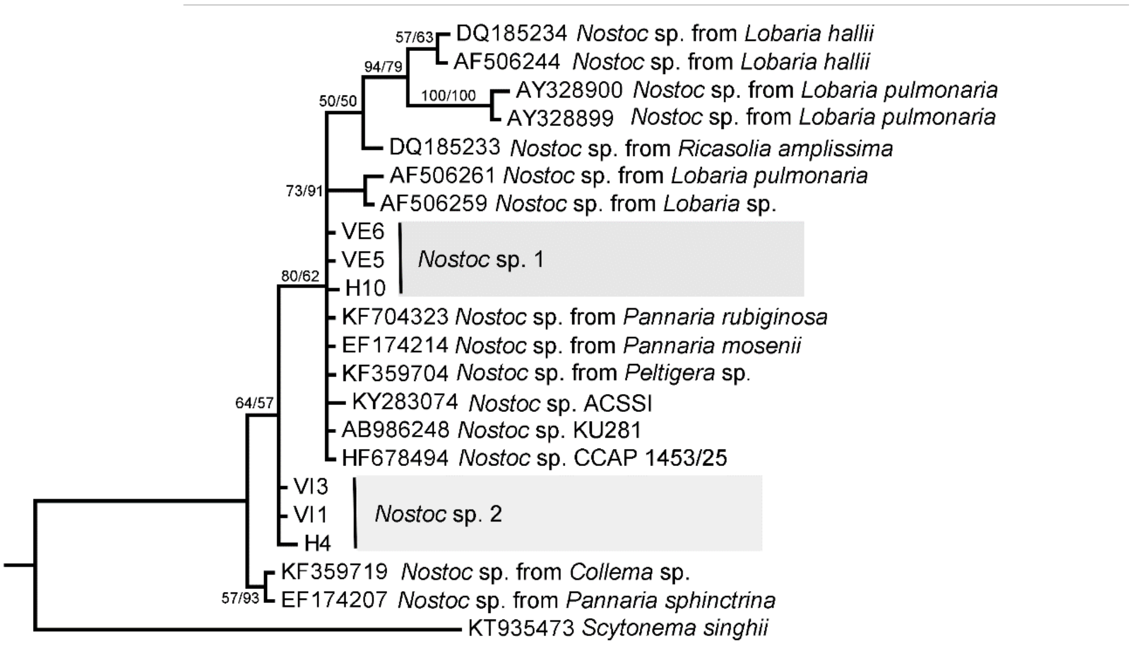

Figure S3. Cyanobiont phylogenetic analysis. Rooted gene tree representing 15 *Nostoc* sequences retrieved from the GenBank and six newly generated sequences (VI1-3, VE5-6, H4-10). Values at nodes indicate statistical support estimated by two methods: posterior probabilities (PP, MrBayes analysis) and bootstrap support (BS, RAxML analysis).

Figure S4

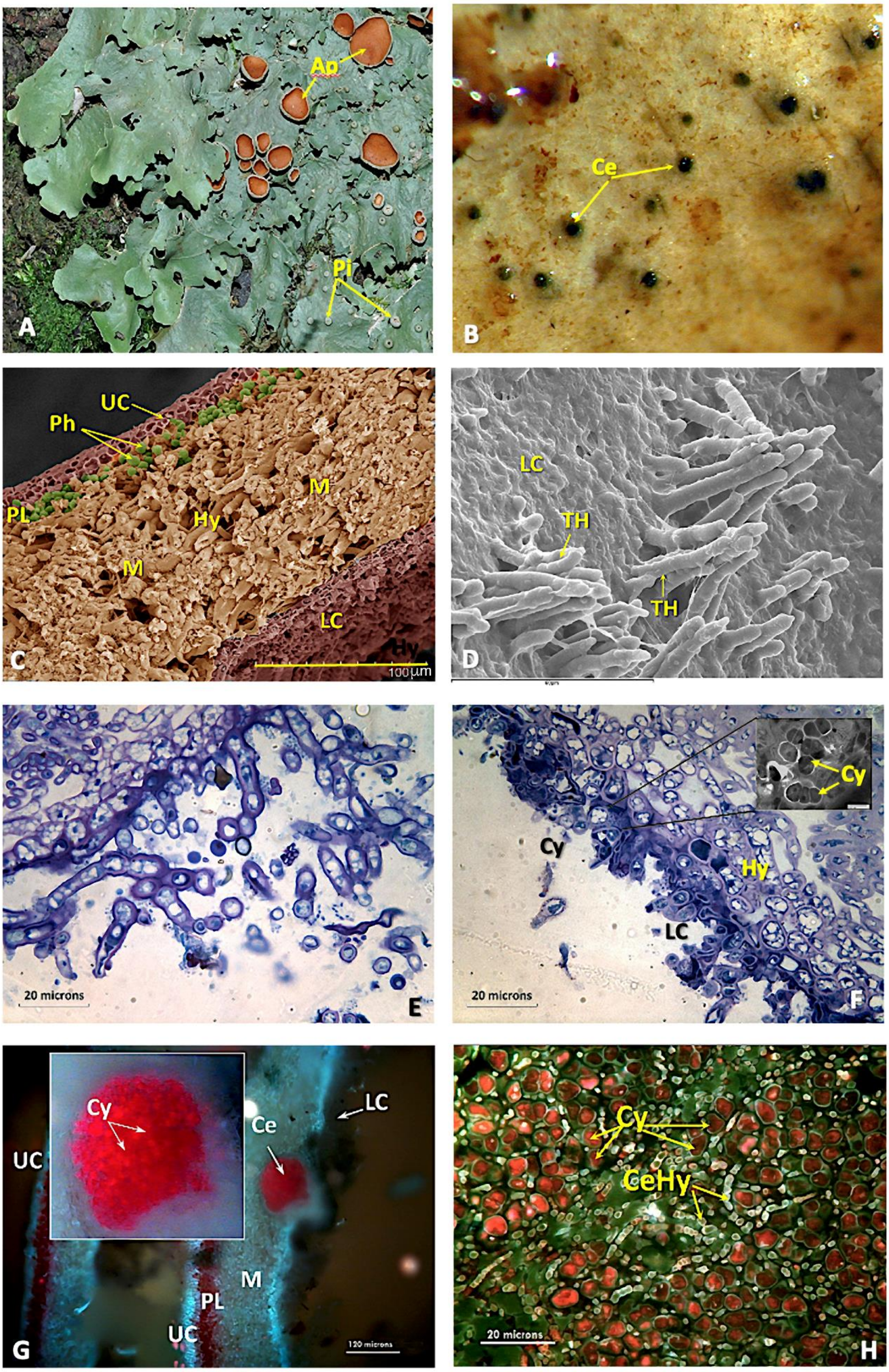
Ap
Pi
A
Ce
B
UC
Ph
PL
Hy
M
M
LC
Hy
100 µm
C
LC
TH
TH
D
20 microns
E
Cy
Cy
Hy
LC
20 microns
F
Cy
UC
Ce
LC
M
PL
UC
120 microns
G
Cy
CeHy
20 microns
H

Figure S4. (A). *Ricasolia virens* thallus habitus with apothecia and pycnidia entering the upper cortex. (B). Picture of the underside of a thallus of *R. virens* showing the beginning of several cephalodia development with the cyanobacteria surrounded by mucilages. (C). False colour LTSEM micrograph showing the internal structure of a thallus of *R. virens* with upper and lower cortex, the medulla and the phycobiont layer. Bar: 100 m. (D). LTSEM micrograph showing the tomentose hyphae growing from the lower cortex. Bar 60 µm. (E). Micrograph of a semi-thin cross-section of a thallus of *R. virens,* stained with toluidine blue, where the paraplectenchyma of the lower cortex is changing to become thinner and more fibrous. Bar: 20 µm. (F). Micrograph of a semi-thin cross-section of the lower cortex of the thallus of *R. virens*, stained with toluidine blue, where some colonies of cyanobacteria are adhering to the lower cortex. The inset shows a TEM micrograph of one of the colonies (see also Figure 10). Bar: 20 µm. (G). Epifluorescence LM micrograph of a cross-section of a thallus of *R. virens*. The red fluorescent emission of cephalodium due to the presence of cyanobacteria is evident. Bar: 120 µm. (H). Epifluorescence LM micrograph of an *R. virens* cephalodium showing cyanobacteria (in red) and cephaloidal hyphae. Bar: 20 µm. Abbreviations: Ap, Apothecia; Ce: Cephalodium; CeHy: Cephalodial Hyphae; Cy: Cyanobacteria; Hy: Hyphae; LC: Lower Cortex; M: Medulla; PL: Phycobiont Layer; Py, Pycnidium; TH, Tomentose Hyphae; UC: Upper Cortex.

Figure S5

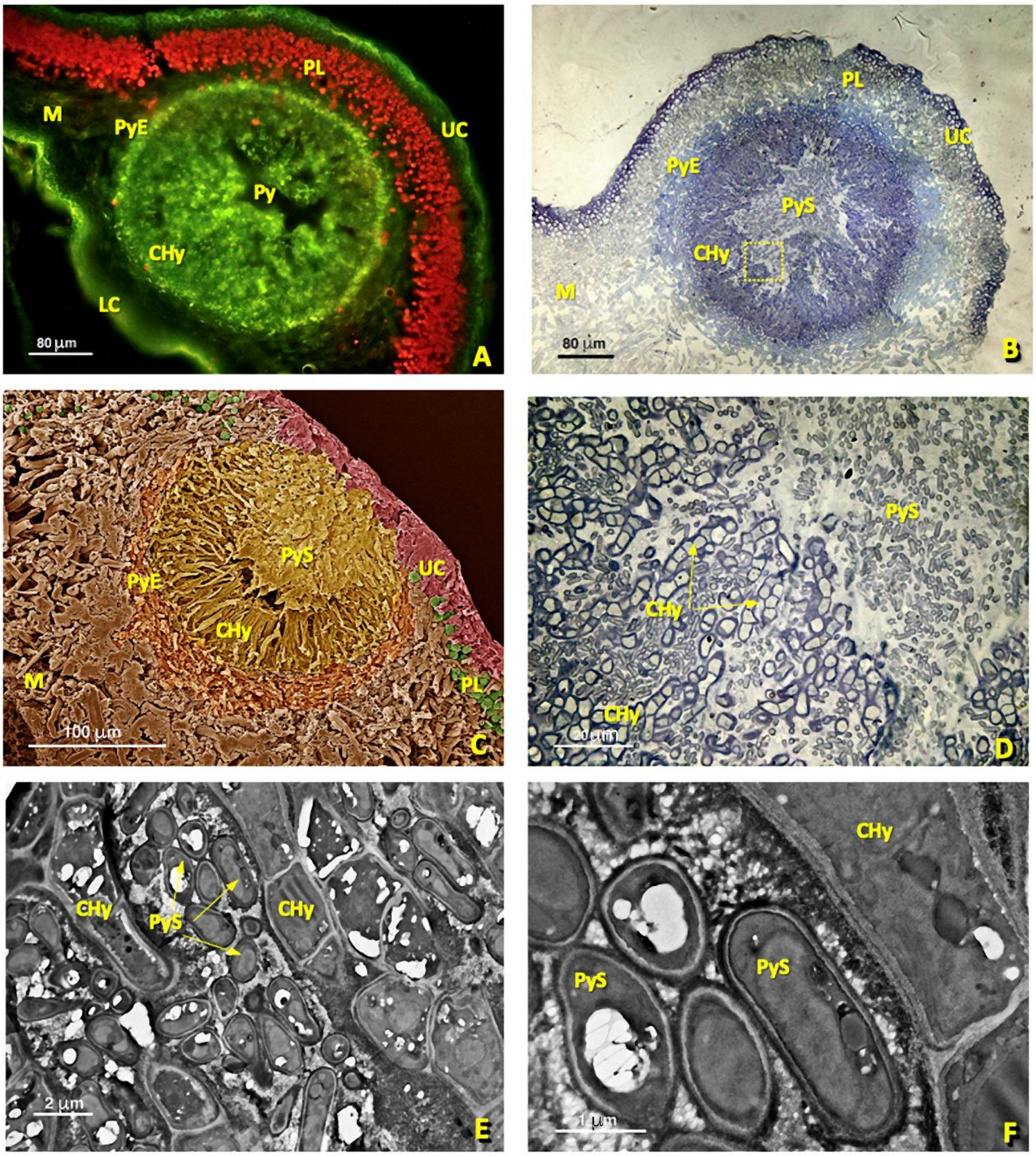

Figure S5. LM micrographs of the pycnidial structures in *Ricasolia virens*. (A) Section by freezing of a thallus seen with UV epifluorescence showing a pycnidium. (B) Semi-thin section of a thallus, stained with toluidine blue, where the structure of a pycnidium is well defined. (C) False colour LTSEM micrograph showing the structure of a pycnidium. (D) LM micrograph of a semi-thin section, stained with toluidine blue, (inset in Figure S5B) of the interior of a pycnidium. Conidiogenous hyphae and pycnidiospores are in view. (E and F) TEM micrograph inside a pycnidium, the ultrastructure of conidiogenous hyphae and pycnidiospores. A and B, bar: 80 µm C, bar: 100 µm; D, bar: 20 µm; E, bar: 2 µm F, bar: 1 µm. Abbreviations: CHy: Conidiogenous Hyphae; LC: Lower Cortex; M: Medulla; PL: Phycobiont Layer; Py, Pycnidium; PyE, Pycnidium Sheath; PyS, Pycnidiospores; UC: Upper Cortex.

Figure S6

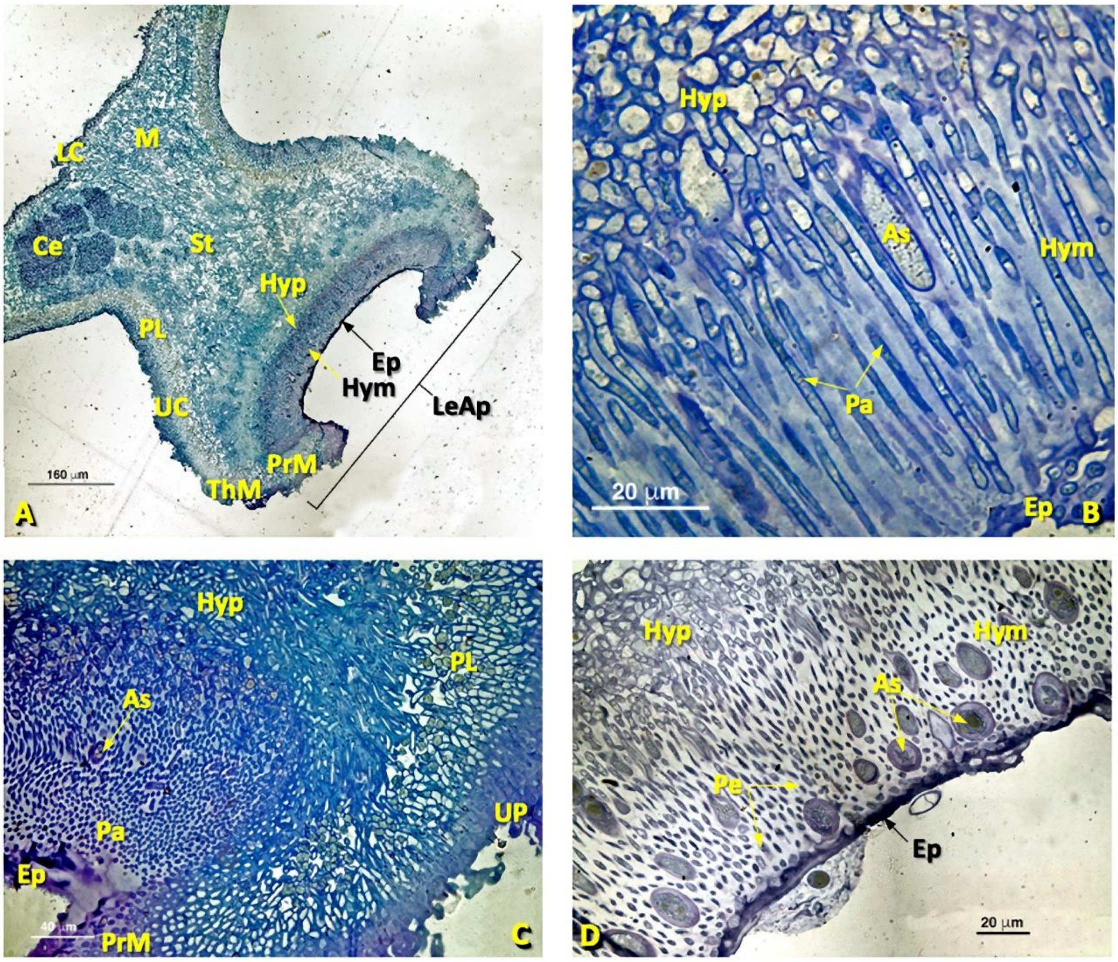

Figure S6. Structures of the zeorin apothecia in *Ricasolia virens*, by LM of semi-thin sections, stained with toluidine blue. (A). Micrograph of a longitudinal semi-thin section of a stipitate zeorin apothecium with a developing cephalodium near the lower cortex. Bar: 160 µm (B, C and D) Micrographs showing some details of the hymenium. Bar: (B and D): 20 µm Bar (C): 40 µm Abbreviations: Am, Amphithecium; As, Ascus; Ce: Cephalodium; Ep, Epithecium; Hym: Hymenium; Hyp, Hypothecium; LC: Lower Cortex; LeAp, Lecanorine Apothecia; M: Medulla; Pa, Paraphyses; PL: Phycobiont Layer; PrM, Proper Margin; St, Stipe; ThM, Thalline Margin; UC: Upper Cortex.
