# Supplementary material for: The Tripartite Lichen Ricasolia virens: Involvement of Cyanobacteria and Bacteria in Its Morphogenesis
Source: Microorganisms. 2023 Jun 7;11(6):1517. doi: 10.3390/microorganisms11061517 (PMC10305269; doi:10.3390/microorganisms11061517)
Supplement: Supplementary file 1 [file microorganisms-11-01517-s001.zip › microorganisms-2403179-Supplementary Tables S1-S3.pdf]

Supplementary Table S1. Specimens used for anatomical studies.

Code	Lat;Lon ED50	Phorophyte	Zone	Height (masl)	Collected by
Vi1	N 43º 26' 10″; W 5º 12' 45,0″	*Ulmus glabra* Huds.	Hayedo de la Biescona[a]	420	*E. Barreno & JS. Álvarez-Aspra*
Vi2	N 43° 20′ 12′′; W 5° 12′ 43,4′′	*Fagus sylvatica* L.	Hayedo de la Biescona[a]	430	*JS. Álvarez-Aspra*
Vi3	N 43° 26′ 11′′; W 5° 12′ 50,0′′	*Fagus sylvatica* L.	Hayedo de la Biescona[a]	442	*JS. Álvarez-Aspra*
Vi4	N 43° 26′ 14′′; W 5° 12′ 46,5′′	*Quercus robur* L.	Hayedo de la Biescona[a]	475	*JS. Álvarez-Aspra*
Vi5	N 43° 26′ 14,2′′; W 5° 12′ 46,0′′	*Quercus robur* L.	Hayedo de la Biescona[a]	477	*JS. Álvarez-Aspra*
Vi6	N 43° 26′ 11,2′′; W 5° 12′ 47,6′′	*Quercus robur* L.	Hayedo de la Biescona[a]	432	*JS. Álvarez-Aspra*
Vi7	N 43° 26′ 14′′; W 5° 12′ 50,5	*Quercus robur* L. y *Hedera helix* L.	Hayedo de la Biescona[a]	460	*JS. Álvarez-Aspra*
Vi8	N 43° 26′ 14,4′′; W 5° 12′ 47,2′′	*Quercus robur* L. y *Hedera helix* L.	Hayedo de la Biescona[a]	484	*JS. Álvarez-Aspra*
Vi9	N 43° 26′ 14,4′′; W 5° 12′ 47,4′′	*Quercus robur* L. y *Hedera helix* L.	Hayedo de la Biescona[a]	484	*JS. Álvarez-Aspra*
Vi10	N 43° 26′ 15′′; W 5° 12′ 46,7′′	*Quercus robur* L. y *Hedera helix* L.	Hayedo de la Biescona[a]	484	*JS. Álvarez-Aspra*
Vi11	N 43° 26′ 15′′; W 5° 12′ 46,7′′	*Quercus robur* L. y *Hedera helix* L.	Hayedo de la Biescona[a]	480	*JS. Álvarez-Aspra*
Vi12	N 43° 26′ 14′′; W 5° 12′ 46,1′′	*Quercus robur* L. y *Hedera helix* L.	Hayedo de la Biescona[a]	480	*JS. Álvarez-Aspra*
Vi13	N 43° 27′ 1,7′′; W 5° 14′ 50,5′′	*Castanea sativa* Miller	Sierra del Sueve[b]	625	*JS. Álvarez-Aspra*
Ve1 a Ve7	N 43° 26' 55′′; W 5° 10' 00"	*Fraxinus excelsior* L.	Vega de Sebarga[c]	206	*V.M. Vázquez & P. Vázquez*
H1 a H4	N 43° 15' 38"; W 6° 53' 01"	*Acer pseudoplatanus* L.	Riberas del Río Ahío[d]	189	*V.M. Vázquez & P. Vázquez*
H5 a H8	N 43° 15' 38"; W 6° 53' 01"	*Quercus robur* L.	Riberas del Río Ahío[d]	189	*V.M. Vázquez & P. Vázquez*
H9 a H12	N 43° 15' 38"; W 6° 53' 01"	*Hedera* hélix L.	Riberas del Río Ahío[d]	189	*V.M. Vázquez & P. Vázquez*

a. Hayedo de la Biescona (Caravia)
b. Sierra del Sueve, la Cordobana (Colunga), near the *Taxus Baccata* L forest.
c. Vega de Sebarga (Amieva). Melon river shores..
d. Riberas del Río Ahío. Pesoz.

Supplementary Table S2. Herbarium University of Valencia (VAL-Lich) Specimens revised.

Code	VAL-Lich	Lat;Lon ED50	Phorophyte	Zone	Height (masl)	Identified by	Date
Heb1	20370	N 43º 01' 56,7''; W 6º 41' 32,3''	*Fraxinus excelsior* L.	Monte Muniellos[a]	690	*S. Pérez-Ortega & S. Fos.*	19/10/2000
Heb2	20606	N 43º 01' 56,7''; W 6º 41' 32,3''	*Quercus petraea (Matt.) Liebl.)*	Monte Muniellos[b]	940	*S. Pérez-Ortega & S. Fos.*	18/10/2000
Heb3	20808	N 43º 01' 56,7''; W 6º 41' 32,3''	*Quercus petraea (Matt.) Liebl.)*	Monte Muniellos[b]	870	*S. Pérez-Ortega & S. Fos.*	18/10/2000
Heb4	20944	N 43º 01' 56,7''; W 6º 41' 32,3''	*Populus canadensis* Moench	Monte Muniellos[a]	690	*S. Pérez-Ortega & S. Fos.*	19/10/2000
Heb5	22102	N 43º 01' 56,7''; W 6º 41' 32,3''	*Acer pseudoplatanus* L.	Monte Muniellos[a]	690	*S. Pérez-Ortega & S. Fos.*	19/10/2000
Heb6	22114	N 43º 01' 56,7''; W 6º 41' 32,3''	*Populus canadensis Moench*	Monte Muniellos[a]	690	*S. Pérez-Ortega & S. Fos*	19/10/2000
Heb7	22434	N 43º 01' 56,7''; W 6º 41' 32,3''	*Fagus silvatica* L.	Monte Muniellos[c]	740	*S. Pérez-Ortega & S. Fos*	20/03/2003
Heb8	25074	N 43º 15' 24.1''; W 01º 49' 02''	*Ulmus glabra* Huds.	Oiartzum[d]	250	*B. Aguirre & G. Renobales*	27/04/1984
Heb9	28937	N 28º 08' 51''; W 17º 17' 46''	*Erica arborea* L.	Valle Gran Rey[e]	1025	*V. Calatayud*	29/07/1998
Heb10	28938	N 28º 07' 20''; W 17º 13' 22''	*Laurus azorica* (Seub.) Franco	Las Mimbreras[f]	975	*V. Calatayud*	29/07/1998

a. Ribereña con plágano atravesada por el río Tablizas. Monte Muniellos (Cangas de Narcea).
b. Camino de Fuenculebrera. Monte Muniellos (Cangas de Narcea).
c. Camino de Pico Luis. Monte Muniellos (Cangas de Narcea).
d. Parque natural de Aiako Harria. Oiartzun (País Vasco)
e. Valle Gran Rey. Raso de D. Pedro. La Gomera
f. Las Mimbreras. La Gomera.

Supplementary Table S3. Location and GenBank accession numbers for collections of *Ricasolia virens* samples used in this study.

Locality	Sample code	ITS Mycobiont	ITS Photobionts	16 S	23SU
Riberas del Río Ahío. Pesoz (Asturias, Spain)	**H4**	OP925492	OP932022	OP925747	OP932028
Riberas del Río Ahío. Pesoz (Asturias, Spain)	**H10**	OP925493	OP932023	OP925748	OP932029
Vega de Sebarga (Amieva). Melón river (Asturias, Spain)	**VE5**	OP925494	OP932024	OP925749	OP932030
Vega de Sebarga (Amieva). Melón river (Asturias, Spain)	**VE6**	OP925495	OP932025	OP925750	OP932031
Hayedo de la Biescona (Caravia) (Asturias, Spain)	**VI1**	OP925496	OP932026	OP925751	OP932032
Hayedo de la Biescona (Caravia) (Asturias, Spain)	**VI3**	OP925497	OP932027	OP925752	OP932033
